# 16S rRNA Gene Diversity in Ancient Gray and Pink Salt from San Simón Salt Mines in Tarija, Bolivia

**DOI:** 10.1128/MRA.00820-20

**Published:** 2020-10-08

**Authors:** Wolf T. Pecher, Fabiana L. Martínez, Priya DasSarma, Daniel Guzmán, Shiladitya DasSarma

**Affiliations:** a Institute of Marine and Environmental Technology, University of Maryland, Baltimore, Maryland, USA; b Department of Microbiology and Immunology, University of Maryland, Baltimore, Maryland, USA; c Yale Gordon College of Arts and Sciences, University of Baltimore, Baltimore, Maryland, USA; d Instituto de Investigaciones para la Industria Química, Consejo Nacional de Investigaciones Científicas y Técnicas, Universidad Nacional de Salta, Salta, Argentina; e Centro de Biotecnología, Faculty of Sciences and Technology, Universidad Mayor de San Simón, Cochabamba, Bolivia; University of Southern California

## Abstract

DNA from 250 million-year-old pink and gray salts from mines in Tarija, Bolivia, subjected to 16S rRNA gene amplicon sequencing and analysis provided evidence for similar but distinct prokaryotic communities. The results constitute a snapshot of archaeal and bacterial microorganisms in these remote and ancient salt deposits.

## ANNOUNCEMENT

Ancient subsurface salt deposits are of interest from the perspective of extremophiles, evolutionary biology, and astrobiology (1, 2). Studies on their microbial diversity are needed for expanding our understanding of adaptation to hypersaline environments, including high-salinity/low-water conditions and UV/ionizing radiation tolerance, and potential habitability on Mars (3–5). To date, relatively few studies have been conducted that address microbial diversity in salt evaporites (6–12). Ancient salt deposits have been identified in the Department of Tarija, Bolivia, that were formed during the Early Triassic epoch ∼250 million years ago and are a source of both pink and gray salt (13). This study provides the first insights into microbial diversity in subsurface Bolivian salt mines.

Samples were collected in April 2010 from the salt mines at San Simón (Burdet O’Connor Province, Department of Tarija, Bolivia). The mines are located at an elevation of 1,230 m above sea level, where temperatures range from 10°C to 37°C. The samples, BOL5 (gray salt) (21°24′29.27″S, 64°07′55.55″W) and BOL6 (pink salt) (21°24′19.73″S, 64°07′51.52″W), were processed as previously described (12, 14, 15). Briefly, samples were collected using flame-sterilized tools and placed into sterile plastic bags. Salt crystals were sterilized with 70% ethanol prior to DNA extraction using PowerLyzer PowerSoil DNA extraction kits (MO BIO Laboratories, Inc., Carlsbad, CA). Library construction and 16S rRNA gene amplicon paired-end sequencing (2 × 300 nucleotides) of the V3 to V4 region was performed on a MiSeq platform per the manufacturer’s recommendations (Illumina, Inc., San Diego, CA) using the primers Bakt_341F and Bakt_805R (16).

Sequencing resulted in 53,325 (BOL5) and 307,289 (BOL6) paired-end raw reads. The raw reads were processed with Mothur v1.44.1 and the sequences analyzed with R v3.6.1 (https://www.mothur.org/wiki/MiSeq_SOP) (17–19). The reads were assembled with a quality score threshold of 20. Sequences longer than 475 bp, those with ambiguities and homopolymers (>8 bp), and chimeras were removed. The remaining sequences (BOL5, 18,333 sequences; BOL6, 74,789 sequences) were aligned against the SILVA small subunit (SSU) Ref NR 99 database v132, and sequences with at least 97% similarity were binned into operational taxonomic units (OTUs) (20). The OTUs were classified with a pseudobootstrap value of 80 against the reference database, trimmed to positions 201 to 1000 of the 16S rRNA gene sequence of Escherichia coli (GenBank accession number J01859.1). Singletons and nonprokaryotic sequences were removed, resulting in 41 (BOL5) and 69 (BOL6) OTUs and 18,194 (BOL5) and 74,460 (BOL6) sequences.

For the BOL5 and BOL6 sequences, 15.67% and 40.62% were classified as *Archaea* and 84.33% and 59.38% as *Bacteria*, respectively. All archaeal sequences were classified as the genus *Halorubrum* (phylum, *Euryarchaeota*) (Table 1). All bacterial sequences were classified at the phylum level. *Proteobacteria* (BOL5, 69.74%; BOL6, 78.99%) was the most prevalent genus, phylum, followed by *Chloroflexi* (17.96%) for BOL5 and *Firmicutes* (9.92%) for BOL6. For BOL5 and BOL6, 54.99% and 66.66% of the sequences, respectively, were classified at the genus level. *Delftia* (17.36%) was the most prevalent genus for BOL5, followed by *Sulfuritalea* (11.34%), and for BOL6, *Anaeromyxobacter* (22.37%) was the most prevalent, followed by *Delftia* (16.47%) (Table 1). These findings represent the first insights into prokaryotic diversity in San Simón gray and pink salts.

**TABLE 1:** Prevalence of archaeal and bacterial 16S amplicons at the phylum and genus levels in gray (BOL5) and pink (BOL6) salt from the San Simón salt mines (Burdet O’Connor Province, Department of Tarija, Bolivia)

Sample and taxonomic category^*a*^	Total no. of sequences	Abundance (%)^*b*^
Gray salt (BOL5)		
*Archaea*		
Phylum		
*Euryarchaeota*	2,851	100
Genus		
*Halorubrum*	2,851	100
*Bacteria*		
Phyla		
*Proteobacteria*	10,701	69.75
*Chloroflexi*	2,755	17.96
*Actinobacteria*	1,439	9.38
Genera		
*Delftia*	2,664	17.36
*Sulfuritalea*	1,740	11.34
*Pseudomonas*	1,529	9.97
*Iamia*	632	4.12
*Pedomicrobium*	592	3.86
*Pseudolabrys*	466	3.04
*Sphingomonas*	340	2.22
*Microbacterium*	248	1.62
*Methylobacillus*	84	0.55
*Corynebacterium*	52	0.34
Pink salt (BOL6)		
*Archaea*		
Phylum		
*Euryarchaeota*	30,245	100
Genus		
*Halorubrum*	30,245	100
*Bacteria*		
Phyla		
*Proteobacteria*	34,927	78.99
*Firmicutes*	4,387	9.92
*Chloroflexi*	3,286	7.43
Genera		
*Anaeromyxobacter*	9,892	22.37
*Delftia*	7,283	16.47
*Pseudomonas*	7,229	16.35
*Ralstonia*	1,859	4.20
*Iamia*	1,501	3.39
*Sphingomonas*	775	1.75
*Anaerolinea*	772	1.75
*Caldalkalibacillus*	101	0.23
*Kroppenstedtia*	28	0.06
*Staphylococcus*	17	0.04

a The 3 most prevalent phyla and 10 most prevalent genera for each sample are shown.

b Abundance was calculated based on the total number of sequences in each domain.

### Data availability.

The 16S rRNA gene amplicon data sets are available at NCBI GenBank under accession numbers SRR12127162 (BOL5) and SRR12127193 (BOL6).
